# Enhancing Effector Jurkat Cell Activity and Increasing Cytotoxicity against A549 Cells Using Nivolumab as an Anti-PD-1 Agent Loaded on Gelatin Nanoparticles

**DOI:** 10.3390/gels10060352

**Published:** 2024-05-21

**Authors:** Dalia S. Ali, Heba A. Gad, Rania M. Hathout

**Affiliations:** 1 Department of Biotechnology, Central Administration of Biological, Innovative Products and Clinical Studies, Egyptian Drug Authority, Giza 11566, Egypt; 2 Department of Pharmaceutics and Industrial Pharmacy, Faculty of Pharmacy, Ain Shams University, Cairo 11566, Egypt; 3 Pharmacy Program, Department of Pharmaceutical Sciences, Batterjee Medical College, Jeddah 21442, Saudi Arabia

**Keywords:** Nivolumab, anti-PD-1, gelatin nanoparticles, A549, gelatin, cancer treatment, cytotoxicity and Jurkat cells

## Abstract

The current research investigated the use of gelatin nanoparticles (GNPs) for enhancing the cytotoxic effects of nivolumab, an immune checkpoint inhibitor. The unique feature of GNPs is their biocompatibility and functionalization potential, improving the delivery and the efficacy of immunotherapeutic drugs with fewer side effects compared to traditional treatments. This exploration of GNPs represents an innovative direction in the advancement of nanomedicine in oncology. Nivolumab-loaded GNPs were prepared and characterized. The optimum formulation had a particle size of 191.9 ± 0.67 nm, a polydispersity index of 0.027 ± 0.02, and drug entrapment of 54.67 ± 3.51%. A co-culture experiment involving A549 target cells and effector Jurkat cells treated with free nivolumab solution, and nivolumab-loaded GNPs, demonstrated that the latter had significant improvements in inhibition rate by scoring 87.88 ± 2.47% for drug-loaded GNPs against 60.53 ± 3.96% for the free nivolumab solution. The nivolumab-loaded GNPs had a lower IC_50_ value, of 0.41 ± 0.01 µM, compared to free nivolumab solution (1.22 ± 0.37 µM) at 72 h. The results indicate that administering nivolumab-loaded GNPs augmented the cytotoxicity against A549 cells by enhancing effector Jurkat cell activity compared to nivolumab solution treatment.

## 1. Introduction

Programmed cell death 1 (PD-1) is an immune checkpoint receptor that plays a crucial role in regulating T cells, monocytes, natural killer cells, and macrophages [1]. Programmed death ligand 1 (PD-L1) is a crucial ligand that binds with PD-1 [2], and is expressed in various types of tumor cell lines, like lung cancer, breast cancer, and melanoma [3].

The interaction between PD-1 and its partner protein, PD-L1, is particularly noteworthy for its ability to prevent autoimmunity while also promoting immune evasion of tumor cells [4,5]. By exerting this inhibitory effect on T cells, PD-1/PD-L1 signaling helps maintain self-tolerance and prevent excessive inflammation or tissue damage resulting from an overactive immune response [6]. However, when cancerous tumors express high levels of PD-LI, it can interfere with the function of anti-tumor lymphocytes, essentially allowing malignant cells to evade detection and destruction by the body’s own defense system [7]. 

**Figure 1:**
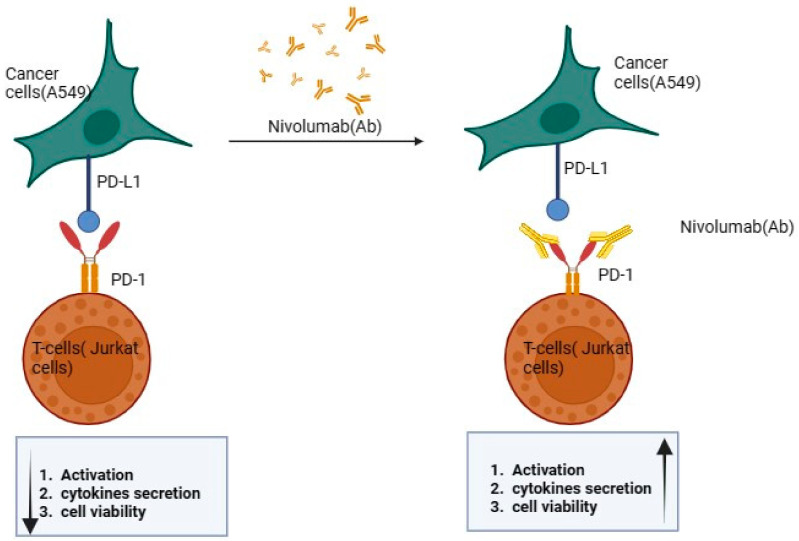
Effect of nivolumab on the PD-1/PD-L1 pathway. Arrows in the figure indicate increase or decrease of the mentioned effect.

The augmentation of PD-1 activity has been linked to the amelioration of immune disorders that are mediated by T cells, indicating a promising avenue for therapeutic intervention. Wu and colleagues recently introduced a new multidisciplinary approach to inhibiting PD-1 and PD-L1 molecules using nanotechnology, aiming to preserve T cell activity in cell therapy [8]. This finding suggests that targeting the PD-1 pathway could provide an effective strategy for managing diseases where aberrant T cell responses play a significant role in pathogenesis [9,10], as illustrated in Figure 1.

In contrast, the opposition or antagonism of PD-1 has demonstrated a significant boost in anti-tumor immunity, indicating that blocking PD-1 can potentially unleash an effective attack on cancer cells by enhancing the T cell response against them [11,12]. Such intricate molecular pathways offer promising insights into new therapeutic strategies for treating complex medical conditions through modulation of this key regulatory checkpoint molecule, PD-1 [13].

The inhibition of PD-1 through antibodies such as nivolumab, an anti-PD-1 immune checkpoint monoclonal antibody (mAb), enhances the conversion of inactive T cells into active effector T cells, thus enabling a more effective immune response against tumors. Despite the significant progress in immunotherapy brought about by immune checkpoint inhibitors, further improvement is necessary to increase their efficacy [14,15]. PD-1 pathway blockade has been identified as a promising treatment strategy for various types of cancer, but it comes with an array of adverse events that can pose serious risks to patients [16]. This is exemplified using anti-PD-1 mAb nivolumab in solitary treatment, which was found to result in only about 50% of patients achieving overall survival over a three-year period [17]. 

The challenges associated with this type of therapy are further highlighted by the increasing incidence of toxicity observed among patients undergoing treatment [8,17]. These toxicities include liver and pancreatic toxicity, inflammatory pneumonitis, and interstitial nephritis, all of which have the potential to be fatal [18,19]. The toxicities associated with the use of immune checkpoint inhibitors are believed to stem from an over-activation of the immune system [20,21,22,23]. However, despite their potential as a promising therapeutic option, one significant drawback is the high dosage required for effective treatment, which incurs exorbitant costs [24]. 

Consequently, researchers have been exploring various strategies to enhance the efficacy of this type of treatment [25]. One such approach involves leveraging biomaterial-based delivery systems like hydrogels [12], microneedle-patch-assisted delivery [26,27], and nanoparticles [27,28,29]. Nanoparticles offer several distinct advantages that make them an attractive candidate for delivering anti-PD1 antibodies: not only do they improve pharmacokinetics and biodistribution, but also intracellular delivery capacity, while boosting tissue penetration levels in vivo [30,31,32].

Gelatin nanoparticles (GNPs) have gained a significant attention as a highly promising biomaterial for the development of advanced delivery systems [33,34]. The use of GNPs offers numerous advantages in biomedical applications, where they can efficiently encapsulate and deliver therapeutic agents such as drugs or genes to targeted sites within the body [35,36,37]. The unique characteristics of GNPs enable them to overcome many limitations associated with conventional drug delivery approaches [38]. GNPs are biocompatible, non-toxic, and biodegradable due to being made from gelatin. They are well tolerated by the body and broken down into non-toxic by-products after drug delivery. GNPs can passively accumulate in tumor tissues, thanks to their size and the enhanced permeability and retention effect. The gelatin network structure within GNPs can control the release rate of loaded drugs by adjusting cross-linking density, responding to the acidic tumor environment for triggered drug release at the tumor site [39,40,41,42]. Moreover, these nanoparticle-based delivery systems can be easily tuned by modifying various parameters such as particle size, shape, surface charge, and functionalization with targeting ligands. Such modifications further enhance their stability in physiological conditions and ensure selective interactions with specific cells or tissues [43]. Furthermore, these tiny carriers facilitate site-specific accumulation within specific tissues, providing better drug concentration at desired locations and thus offering opportunities for cost reduction through lower doses of anti-PD-1 mAbs administration compared to conventional systems. As such, researchers in recent years have devoted considerable effort to investigating ways to improve the efficacy of immune checkpoint inhibitors while minimizing their negative impact on patient health.

Previous studies have demonstrated that loading nivolumab into various nanocarriers can significantly enhance its efficacy [44,45,46]. For instance, the utilization of a metal–organic framework for loading nivolumab has led to improved tumor-specific recognition and successful tumor-targeted delivery of agents [46,47]. Additionally, the combination of doxorubicin-loaded PEGylated poly(lactide-co-glycolide) nanoparticles with nivolumab has been shown to safely enhance therapeutic efficacy in a melanoma model, showcasing promising potential for improving treatment outcomes [48]. Another study aimed to reverse resistance to PD-1 inhibitors by using nanoparticle albumin-bound (nab) paclitaxel in combination with nivolumab as the treatment of recurrent or metastatic head and neck squamous-cell carcinoma (RM-HNSCC), which progressed on a PD-1 inhibitor [49]. Incorporating nivolumab into nanoparticles can also enhance its efficacy against cancer cells while minimizing off-target effects, as demonstrated in studies with other drugs like imatinib mesylate [50].

In line with this goal, the present study aimed to explore whether encapsulating the anti-PD-1 antibody nivolumab within GNPs could enhance its effectiveness against lung cancer cells without compromising safety. Drug-loaded GNPs were prepared with the optimization of independent factors such as the amount of cross-linker used, cross-linking times, and stirring speeds. Full in vitro characterization was performed. Finally, to test the effectiveness and safety of the optimized GNPs, experiments using co-cultures consisting of A549 cell lines and Jurkat cells were conducted.

## 2. Results and Discussion

### 2.1. Particle Size, Zeta Potential, and Polydispersity Index of the Prepared Plain GNPs

The impact of nanotechnology on the advancement of drug delivery systems is heavily influenced by both PS and PDI, as they have a direct effect on biodistribution, targeting characteristics, toxicity levels, and the fate of these nano-delivery systems [51]. Certain factors need to be considered for successful synthesis of GNPs. These factors include the concentration of gelatin, the pH level of the solution prior to desolvation, the type and amount of the used desolvating agent, the rate at which desolvation occurs, and the type and amount of crosslinking agent employed during subsequent steps. Table 1 shows the effect of the different formulation variables on the PS, PDI, and zeta potential of the prepared plain GNPs.

The impact of increasing the percentage of GA from 25% to 50% generally leads to a decrease in PS from 257.5 ± 11.47 nm to 158.6 ± 1.08 nm, while maintaining cross-linking time for 8 h. However, the effect of increasing the GA percent was not pronounced with a cross-linking time of 16 h. This outcome can be attributed to the NPs cross-linking provided by a high GA percentage, which effectively led to sufficient intra-particular amide bonding within the GNPs. Consequently, this enabled satisfactory hardening and shrinkage of the particles. Additionally, because of the short duration of cross-linking time employed, there were no occurrences of inter-particular bridging or aggregation among particles [52].

Conversely, there is an opposite trend observed regarding cross-linking time, where increasing the cross-linking time was associated with an increase in PS of the prepared GNPs. This can be explained by allowing enough time for inter-particular amide bridging between different GNPs due to the symmetrical cross-linking effect of GA, resulting in particle growth through GNP aggregation [53]. Regarding the stirring speed effect, it was found to be dependent on cross-linking time and GA%. The rate at which the gelatin solution is stirred has a significant impact on the formation of homogenous nanoparticles, as vigorous stirring leads to an increase in PS and facilitates the aggregation of gelatin molecules [34]. Regarding the PDIs, they ranged from 0.027 ± 0.02 to 0.302 ± 0.03. It is observed that PDIs are relatively low, indicating a narrow distribution of PS [52,54].

Zeta potential, a measure of the electric charge on the particle surface, has long served as a fundamental indicator of dispersion stability. It provides crucial insights into the tendencies of particles to either aggregate or undergo deposition in various fluid systems [55]. High stirring speeds, as in GNP5, can significantly impact the stability of gelatin nanoparticles and their zeta potential charges. At high stirring speeds, shear forces can cause aggregation, decreasing stability and affecting the zeta potential charges with implications for drug delivery systems [43,56]. Nanoparticles formulations with a high zeta potential could inhibit aggregation [57]. GNPs were found to be positively charged as the molecules were positively charged due to the preponderance of ammonium groups, −NH^3+^ [58,59]. The obtained ZP values together with the small PDI values reflect the effect of the particles’ charge in marinating their stability. As a result, GNP2 has been selected for further experimentation with regards to PS (158.60 ± 1.08 nm), PDI (0.041 ± 0.01), and ZP (21.7 ± 0.15 mV).

### 2.2. Characterization of Nivolumab-Loaded GNPs

#### 2.2.1. Determination of Entrapment Efficiency (%EE)

Nivolumab was successfully encapsulated within the optimized GNPs, achieving an average entrapment efficiency of 54.67 ± 3.51%. The moderate entrapment efficiency of nivolumab can be attributed to its hydrophilic nature, in addition to the loading procedure, which ensured that nivolumab became an indispensable component firmly bound within both the structure and composition of GNPs [37]. However, it is worth noting that there is a tendency for leakage to occur in aqueous environments during or after nanoparticle preparation [60].

#### 2.2.2. Particle Size and Polydispersity Index Measurements

The particle size measurement yielded an average value of 191.90 ± 0.67 nm with a low polydispersity index value of 0.027 ± 0.02 for the nivolumab-loaded gelatin nanoparticles, which indicates a homogeneous size distribution and successful drug entrapment. Such homogeneity is crucial for ensuring consistent therapeutic effects and predictability of the pharmacokinetics cellular uptake, drug release profiles of the nanoparticles, and biodistribution of the nanoparticles. The lack of a significant difference at *p* < 0.05 between the plain and drug-loaded GNPs suggests that the drug loading process did not significantly alter the physical properties of the GNPs, which is desirable in maintaining the original advantageous characteristics of the nanoparticle carrier system. The following study highlights the significance of controlling particle size and PDI in the development of gelatin-based nanocarriers. In “Biopolymer based nanomaterials in drug delivery systems: A review”, the authors discuss various biopolymer-based nanomaterials, including proteins and polysaccharides like gelatin. This review describes the new trends of utilizing biopolymer nanoparticles, such as gelatin, in targeted drug delivery, and highlights the role of particle size and PDI in achieving successful delivery to specific tissues [61].

### 2.3. In Vitro Release Study of Nivolumab from the Prepared GNPs

**Figure 2:**
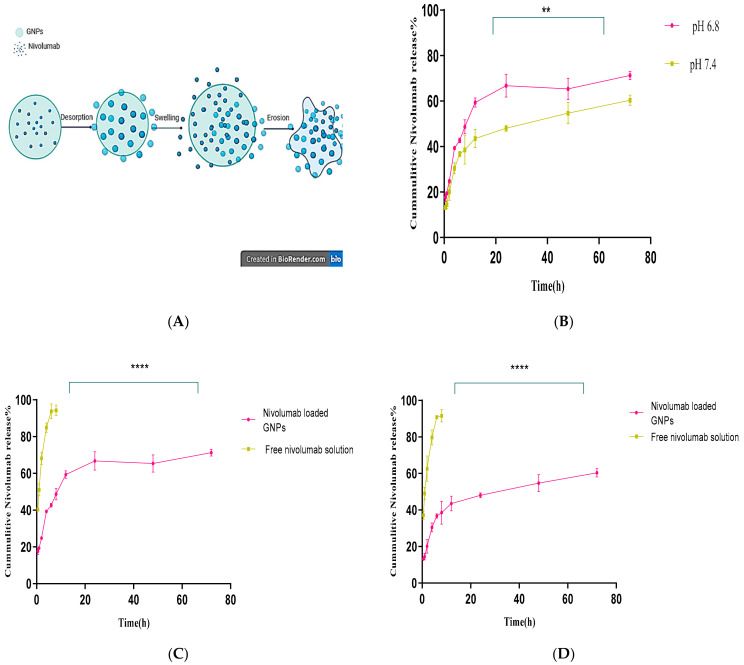
(**A**) Illustrative diagram of drug release mechanism from gelatin nanoparticles. (**B**) Comparison of the cumulative drug release from gelatin nanoparticles at two different pH values (*p* = 0.0012, n = 3). (**C**) Comparison of the cumulative nivolumab release from gelatin nanoparticles and nivolumab solution at pH 6.8 (*p* < 0.0001). (**D**) Comparison of the cumulative nivolumab release from the gelatin nanoparticles and nivolumab solution at pH 7.4 (*p* < 0.0001). ** indicates very significant while **** extremely significant.

The drug release mechanism from nivolumab-loaded GNPs in an in vitro setting involves several intricate processes. Firstly, the drug diffuses through the surrounding medium as it gradually permeates into its surroundings (desorption). Additionally, swelling of hydrophilic or amphoteric matrices such as gelatin usually occurs. Lastly, a “decomposition over time” process occurs and contributes to the sustained release of the encapsulated drug partially or completely during this process (erosion) [52,62,63], as illustrated in Figure 2A. The release profile of nivolumab from the selected formula of GNPs at pH 7.4 (physiological pH) and at a slightly acidic pH 6.8 (cancer cells) is shown in Figure 2B. It is observed that there is an initial burst within the first 30 min in the two studied pHs; however, the rate of drug release is significantly faster (*p* = 0.0011) in an acidic medium. Furthermore, it demonstrates sustained drug release properties with an accumulated drug release percentage of 71.33 ± 1.86% after 72 h in the acidic pH.

Conversely, under physiological pH (7.4), a lower but still noticeable release percentage was recorded of 60.34 ± 2.28% [64,65]. Figure 2C,D shows the release patterns of nivolumab from free nivolumab solution and nivolumab-loaded GNPs in the first four hours. It was observed that the release rate of free nivolumab was remarkably rapid, with approximately 84.87 ± 2.63% (pH 6.8) and 79.73 ± 4.13% (pH 7.4) of nivolumab released. Conversely, for nivolumab-loaded GNPs, only 39.33 ± 0.77% and 30.47 ± 2.44% of nivolumab were released when immersed in media solutions with pH values of 6.8 and 7.4, respectively. This finding indicates that GNPs offer an advantage in drug delivery systems due to their pH sensitivity, which can be utilized for cancer therapy. They exploit the slightly acidic environment of cancer cells (typically with a pH around 6.8) to release their drug payload more actively at the tumor site than in normal tissues (with a neutral pH around 7.4) [66,67]. This pH-dependent release mechanism can enhance the bioavailability of anticancer drugs where they are needed most, potentially improving treatment efficacy and reducing side effects on healthy cells. These properties make gelatin nanoparticles excellent candidates for targeted drug delivery applications in cancer treatments. Such a strategy aims to improve therapeutic outcomes by ensuring higher drug concentrations at the tumor site while minimizing systemic exposure, thereby sparing normal, healthy cells from chemotherapy drugs’ cytotoxic effects [64,65].

### 2.4. Transmission Electron Microscopy (TEM)

**Figure 3:**
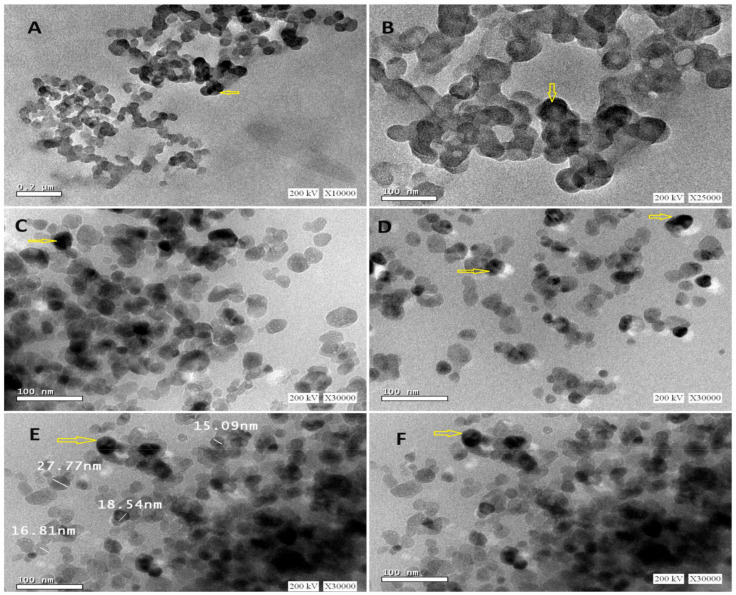
Transmission electron microscopy imaging of nivolumab-loaded GNPs using a voltage of 200 kV and magnification ×10,000 (**A**), ×25,000 (**B**), and ×30,000 (**C**–**F**). The arrows points to some spherically imaged nanoparticles.

According to the findings depicted in Figure 3, examination of the nivolumab-loaded GNPs using TEM demonstrated their homogenous and spherical morphology without any aggregation. Previous studies highlight the significance of nanoparticle morphology and distribution for efficient drug delivery. Spherical nanoparticles are preferred for systemic administration due to their vascular dynamics and cellular uptake characteristics [68]. Homogeneously sized nanoparticles exhibit uniform pharmacokinetics and biodistribution profiles crucial for the desired therapeutic outcome [69], so homogeneity is linked to improved dosing accuracy and therapeutic effects. The spherical structure and non-aggregation are essential for good colloidal stability, maintaining functional attributes in biological environments. Aggregated nanoparticles can lead to reduced efficacy and rapid clearance from the bloodstream. Lack of aggregation supports potential enhanced circulation times and improved tumor targeting for gelatin nanoparticles [70]. The size, shape, and surface characteristics of nanoparticles influence cellular interactions, affecting uptake by different types of cells. Spherical nanoparticles, like nivolumab-loaded gelatin nanoparticles, are easily taken up by cells, enhancing drug delivery efficiency [58]. The physical stability of nanoparticles predicts their behavior in vivo, with homogeneous and stable nanoparticles being suitable for clinical applications to maintain drug release profiles and targeting abilities. Additionally, PS measurements obtained through dynamic light scattering (DLS) showed a good correlation with the PS obtained using TEM, with the exception of the DLS-measured hydrodynamic diameter, leading to slightly larger particles [71]. It is important to note that DLS tends to be sensitive towards detecting the formation of aggregates and favors measurement bias toward larger particles due to its intensity-based nature [52].

### 2.5. Determination of the Binding Activity and Potency of Free Nivolumab and Nivolumab-Loaded GNPs

The results of the three independent ELISA studies revealed that the nivolumab monoclonal antibody (mAb), released from GNPs, maintains its stability after formulation. Additionally, it was observed that the mAb retains its binding activity to Mice PD-1/Fc Chimeras when compared to the drug’s binding activity. This was demonstrated by measuring both the EC50 values of the nivolumab-loaded GNPs and free nivolumab solution. These findings indicate that the formula has preserved and retained binding activity to the coated receptor PD-1, which is comparable to the reference drug.

**Figure 4:**
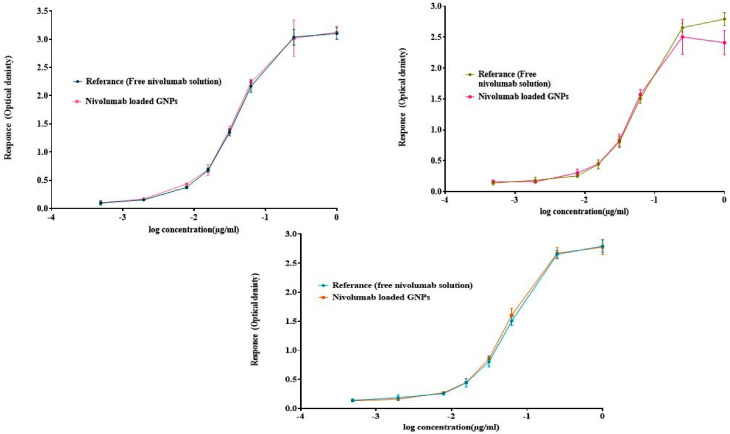
ELISA results of asymmetrical dose–response curves of the three independent runs by plotting log concentration against response using the five-parameter log-logistic equation. Error bars depict means ± SD.

According to Figure 4, the sigmoidal-shaped dose–response curves show the response going up as the concentration increases and the EC_50_ values were broadly found to be in agreement with the reference, which reflects the affinity of the loaded drug to the target receptor. As inferred from Table 2,the binding activity of nivolumab-loaded GNPs to Mice PD-1/Fc Chimeras for the three independent runs was 99.42%, 95.42%, and 92.14%, respectively, compared to the free drug, with a hillslope ratio of 2.698, 2.558, and 2.06 as illustrated in Figure 4, respectively. The slope ratio describes the steepness of the curve, as a slope of 1.0 represents a standard curve; a hillslope greater than 1 represents a steeper curve, which indicates high potency [72].

The S value represents the symmetry parameter, in which S = 1 indicates the symmetry of the curve. If S is greater than 1.0, then the curve is asymmetric, as shown below with S values of 0.371, 0.477, and 0.656, respectively, which represent the five-parameter logistic equation. The top and bottom parameters describe the values at which the curve reaches the plateau. R^2^ represents the goodness of the fit, with R^2^ of 0.997, 0.995, and 0.997, which indicates that the curve comes close to the data points. When comparing the two curves of the sample against the reference to determine parallelism using the F-test when alpha was equal to 0.05, it was found that the results for the three independent runs had *p*-values of 0.509, 0.498, and 0.084, which are greater than the significance level (0.05); therefore, the assays passed the parallelism test, which indicates that the relative potency estimate is valid.

**Figure 5:**
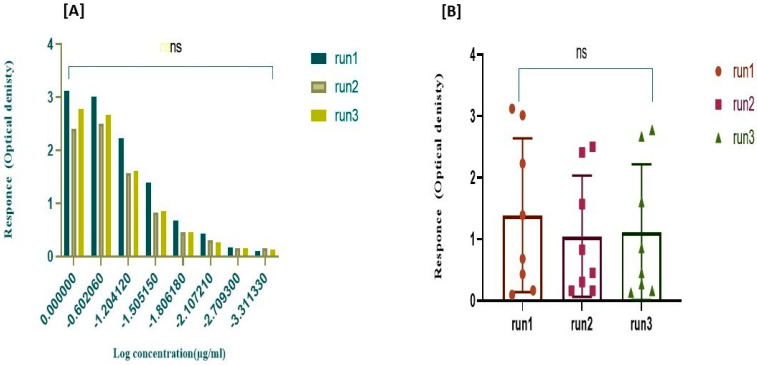
Consistency of the relative potency among the three independent runs of the selected nivolumab-loaded GNPs. (**A**) Comparison of the three runs using F-test when F (10, 57) = 3.489 × 10^−8^), with *p* > 0.999 to demonstrate the parallelism among the curves. (**B**) Comparison of three runs using two-way ANOVA (alpha = 0.05) to demonstrate the non-significant difference between the mean of the responses of the three runs with *p*-value > 0.999. NS: No significance.

The lack of fit was calculated for the three runs with a *p*-value of 0.163, 0.709, and 0.985, as a *p*-value larger than the significance level (0.05) suggests that the residuals pass the normality test. To ensure the method’s consistency in preparing GNPs and establish its replicability, it is vital to compare the binding activity and relative potency among the three runs using statistical analysis. A two-way ANOVA was conducted with alpha set at 0.05. Again, the *p*-value exceeded the *p* > 0.9999 threshold for significance testing. As demonstrated in Figure 5, the statistical analyses indicate no significant difference between the three runs.

### 2.6. In Vitro Cytotoxicity of Gelatin Nanoparticles on A549 Cell Line

**Figure 6:**
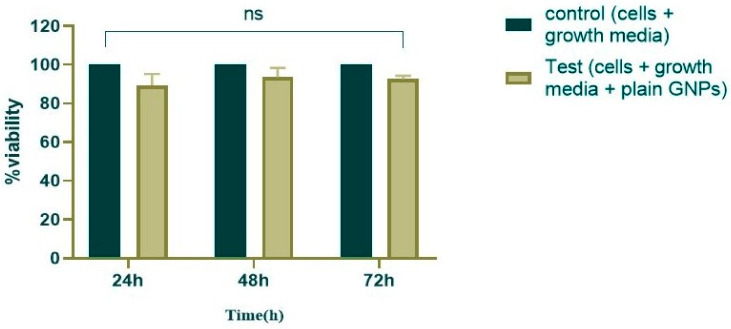
Comparison of the growth and cell viability of plain gelatin nanoparticles against control cells (*p* value = 0.2397), with the interaction considered non-significant (alpha = 0.05). NS: No significance.

The results of the experiment showed that the non-loaded GNPs did not exhibit any significant cytotoxicity effect on the cell culture system of A549 in comparison to the control cells (cells only), which had 100% viability. This finding suggests that the nanoparticles possess a favorable biosecurity profile for further study, which is further illustrated in Figure 6.

### 2.7. Determination of IC_50_ of Free Nivolumab and Nivolumab-Loaded GNPs on the A549 Cell Line

**Figure 7:**
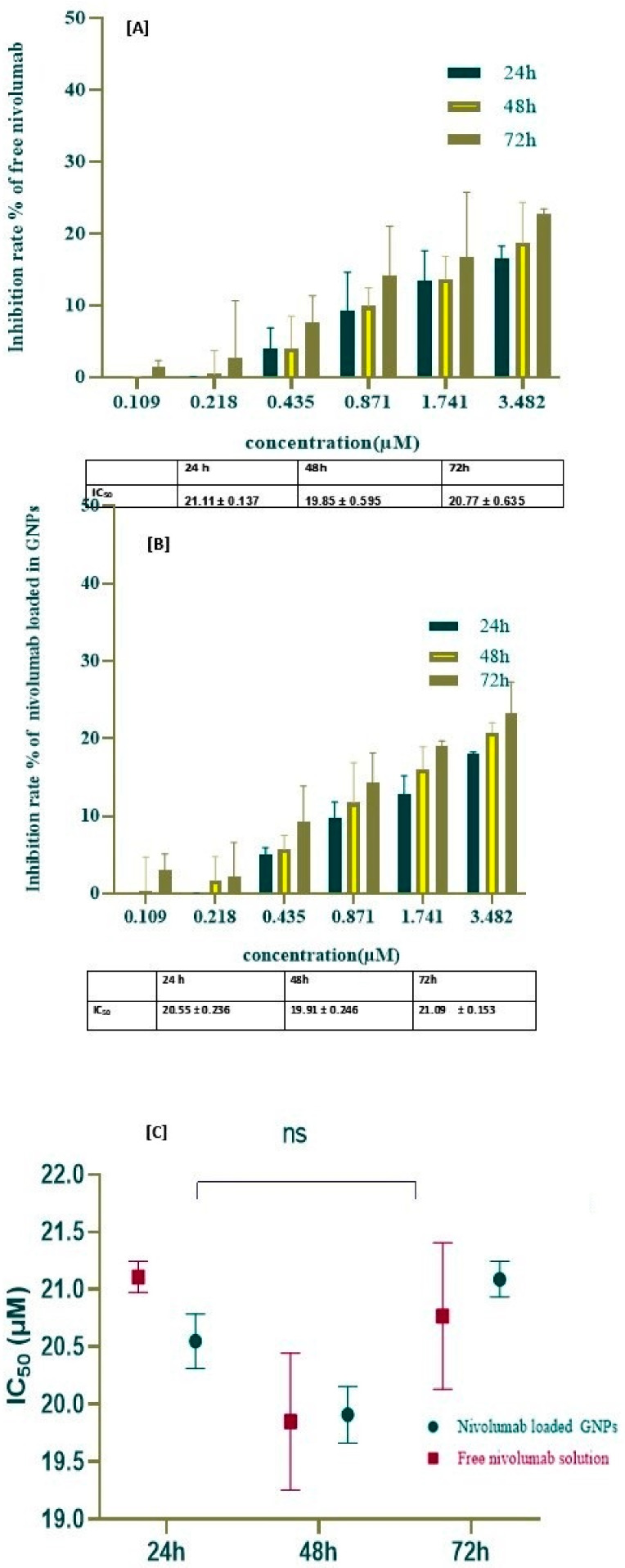
The cytotoxcity of the A549 cell line using an MTS assay and determination of IC_50_ using the non-linear regression normalized dose inhibitory response. (**A**) The inhibition rate of nivolumab-loaded GNPs with different concentrations at different time intervals with a non-significant decrease in IC_50_ (ns = 0.997). (**B**) The inhibition rate of free nivolumabwith different concentrations at different time intervals with a non-significant change in IC_50_ (ns > 0.999). (**C**) The histogram of IC_50_ values of free nivolumab and nivolumab-loaded GNPs at different time intervals (ns = 0.177) compared to the control group.

To further clarify the possible underlying mechanism behind the cell apoptosis in the A549 cell line, an investigation of the inhibitory effects of the free nivolumab and nivolumab-loaded GNPs was performed. Using the MTS assay, the cell line was treated with varying concentrations of nivolumab ranging from 3.482 µM to 0.109 µM through a two-fold dilution at different time intervals: 24, 48, and 72 h. Figure 7A,B, illustrates the inhibition rate% against the concentration to determine the IC_50_s values for free nivolumab and nivolumab-loaded GNPs.

The results indicate that as the incubation period increased, there was a slight dose-dependent increase in the inhibitory effect of nivolumab-loaded GNPs when compared to the control group without a significant difference. In Figure 7A,B, at 72 h, the highest inhibition rate was approximately 23.23 ± 4.021% for nivolumab-loaded GNPs and 22.68 ± 0.75% for the free nivolumab. The findings revealed that there is almost no variation in the maximum inhibition rate, regardless of the differences in the incubation time, with no significant difference in the IC_50_, as illustrated in Figure 7A,B. In contrast, in Figure 7C, the experimental findings clearly exhibit an insignificant change in the IC_50_ of nivolumab-loaded GNPs as compared to free nivolumab. The findings indicate that the nivolumab monoclonal antibody alone did not have a substantial impact on the A-548 cell line. However, there is evidence to suggest that nivolumab can augment the anti-tumor activity of immune cells by promoting the development of an immune response against tumors. Specifically, it has been shown to increase T cell cytotoxicity, thereby potentially improving overall anti-tumor immunity.

### 2.8. Determination of the Optimum Concentration of Jurkat Cells and the Optimum Incubation Period Used in Co-Culture

**Figure 8:**
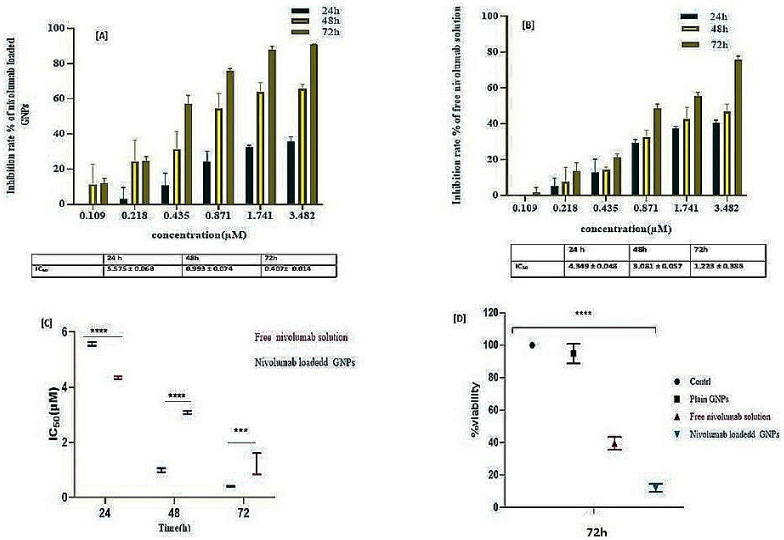
The proliferation of A549/Jurkat cells is determined by MTS assay. (**A**) The IC_50_ of nivolumab-loaded GNPs showed a significant decrease at various time intervals. (**B**) Displays a significant change in IC_50_ for free nivolumab solution at different concentrations and time intervals. (**C**) Shows a significant difference (**** *p* < 0.0001, *** *p* = 0.0007) between the loaded and free nivolumab. (**D**) Displays a significance difference (**** *p* < 0.0001) when comparing the % viability of nivolumab-loaded GNPs with free nivolumab solution using one-way ANOVA.

The Jurkat cell line, a human T lymphocyte immortalized cell line [73], has been found to exhibit elevated expression levels of PD-1 when compared to other immune checkpoint receptors. These findings suggest that the use of Jurkat cells can serve as an efficient tool for exploring the interaction between PD-1 and PD-L1 on tumor cells and aid in planning effective cancer immunotherapies by blocking the PD-1/PD-L1 pathway. In co-culture systems involving direct interactions between A549 and Jurkat cells, the blockade of PD-1/PD-L1 enhanced cytokine production by T cells [74]. This provides valuable insights into how these proteins interact within the context of different physiological conditions, while also helping to understand their roles in suppressing anti-tumor responses [75]. The present study expanded its scope to evaluate the cytotoxic effects of nivolumab-loaded GNPs on A549 target cells with different incubation periods when compared with free nivolumab. The findings revealed that a co-culture ratio of E/T (1:10) [76] and a prolonged 72 h incubation period proved to be optimal conditions, resulting in maximum target cell cytotoxicity of 91.163 ± 0.190, as illustrated in Figure 8A, and a 75.895 ± 1.778 inhibition rate for a similar free drug, as illustrated in Figure 8B.

The IC_50_s of the loaded and free nivolumab decreased with increasing time. Additionally, nivolumab-loaded GNPs demonstrated a significantly (*** *p* = 0.0007) lower IC_50_ value of 0.407 ± 0.014 µM when compared to the free drug (1.223 ± 0.368 µM) at 72 h, as illustrated in Figure 8C. This indicates that the GNPs have improved cellular uptake; the lower the IC_50_, the more potent the drug.

With the exception of the 24 h time interval, the IC_50_ of the free drug (4.349 ± 0.047 µM) was lower than that of the drug-loaded GNPs (5.575 ± 0.068 µM). This may be due to the fast release of the free drug when compared to the loaded form. Rapid burst release is one of the fundamental problems of anticancer therapy, leading to toxicity. In Figure 8D, it is illustrated that nivolumab-loaded GNPs have a lower % viability of 12.12 ± 2.47% when compared to the free nivolumab, of 39.48 ± 3.98%, revealing a significant difference (*** *p* < 0.0001) in the % viability of the selected formulation at a concentration of 500 µg/mL and the same concentration of free nivolumab at the optimum incubation period of 72 h.

### 2.9. Phenotypic Characteristics of Cell Lines

**Figure 9:**
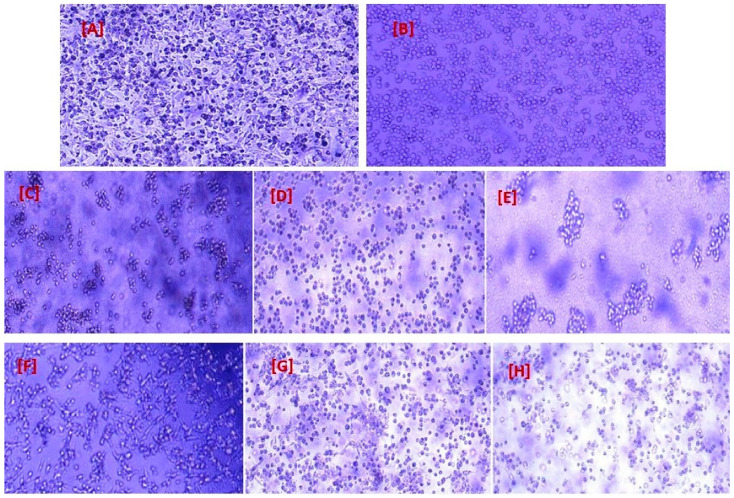
(**A**) A549 cell line subculture with RPMI with glutmax media as control cell. (**B**) Bright field images of Jurkat cells. (**C**) Representation of A549 and Jurkat cell lines treated with nivolumab-loaded GNPs after 24 h; (**D**) cells after 48 h; (**E**) cells after 72 h. (**F**) Image representative of A549 and Jurkat cell lines treated with free nivolumab after 24 h; (**G**) cells after 48 h; (**H**) cells after 72 h.

Figure 9A depicts the A549 epithelial cell line derived from lung cancer, as observed under a microscope. The cells were subcultured two to three times per week using RPMI with glutmax media. By comparison, Figure 9B illustrates Jurkat cells after undergoing subculturing. The base medium utilized for this cell line was RPMI-1640 medium. As part of the maintenance routine, fresh medium was added every 2 to 3 days in accordance with the cell density levels. Figure 9 middle (C, D and E) and lower panels (F, G and H) illustrate the co-culture of two distinct cell lines, exploring the impact of the conjugated drug with GNPs and the free drug, respectively, over varying time intervals of 24, 48, and 72 h. These experimental observations lead to morphological changes exhibited by cells as their viability diminishes over time. Consequently, cells struggle to maintain their typical cellular structure due to the influence exerted by both the drug itself and its encapsulation within GNPs. Thus, findings of the study are noteworthy, as they demonstrate that exposure to nivolumab resulted in morphological alterations in A549 and Jurkat cells, providing visual evidence of the cellular changes induced by these treatments. This has important implications for understanding the mechanisms underlying these interventions and suggests potential avenues for future research into their clinical applications.

### 2.10. Stability

**Figure 10:**
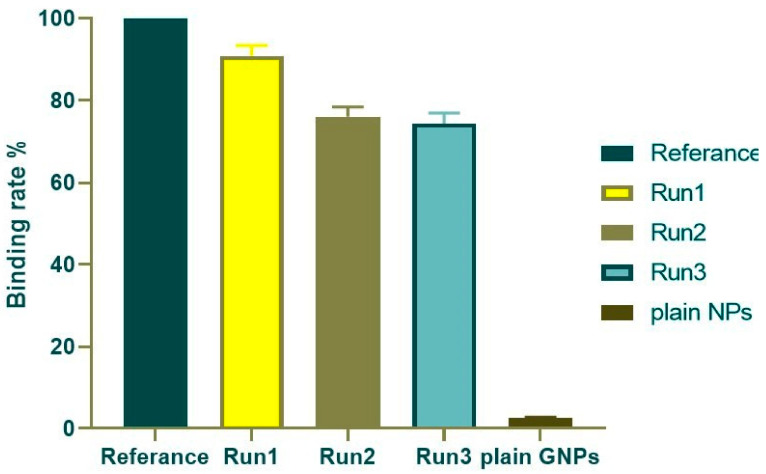
ELISA results of binding rate % of the loaded GNPs at different time intervals against the free nivolumab solution.

This study aimed to evaluate the long-term stability of the selected formulation (GNP2) containing 0.5 mg/mL nivolumab within GNPs after storage for one, three, and six months. For such products, in which the active components are typically proteins, maintenance of molecular conformation and, hence, biological activity, is dependent on non-covalent as well as covalent forces. The products are particularly sensitive to environmental factors such as temperature changes, oxidation, and light [46]. According to Figure 10, the binding rates were found to be 90.912% ± 2.552, 76.089 ± 2.383%, and 74.365 ± 2.563% after one, three, and six months of storage, respectively, when compared to the free drug. Furthermore, the R^2^ value was 0.997, indicating a strong association between the curve fit and the data points. The results indicated that the prepared particles were stable over a period of six months, with preserved binding activity even during storage. This suggests that the formulated particles hold great promise for further exploration and potential use in various applications requiring sustained stability.

## 3. Conclusions

Nivolumab, an anti-PD-1 drug, was successfully loaded on gelatin nanoparticles. The nanoparticles demonstrated a spherical and symmetrical size distribution with a low polydispersity index associated with confirmed stability. In vitro release studies revealed that nivolumab-loaded GNPs exhibited slow and sustained release compared to free nivolumab. Cytotoxicity analysis conducted against the A549 cell line in the presence of the Jurkat cell line revealed the higher effectiveness of the optimum formulation in restoring Jurkat cell activation through blocking the PD-1 and PD-L1 pathways when compared to free nivolumab. GNPs could be a promising carrier for checkpoint inhibitors due to their unique properties, allowing for optimized dosing regimens with reduced toxicity profiles compared to traditional cancer therapy, which often leads to uncontrolled drug dispersion throughout tissues and rapid release, resulting in harmful effects. As a future perspective, in vivo tests can be conducted to further prove the success of the newly developed system as an effective and safe lung cancer therapy. Nanoparticles loaded with nivolumab represent a promising advance in oncology, with the potential to fine-tune immunotherapy and enhance anti-tumor responses. The ongoing research and development in this realm of nanomedicine are addressing challenges and harnessing these advantages to bring forth innovations that could revolutionize cancer therapy.

## 4. Materials and Methods

### 4.1. Materials

Gelatin type A from porcine skin (molecular weight 50–100 kDa, bloom 300), glycine, glutaraldehyde solution grade II (25% in H_2_O), phosphate buffered saline tablets, acetone, dimethyl sulfoxide (DMSO), trypan blue dye, and fetal bovine serum were purchased from Sigma Chemical Co., St. Louis, MO, USA. Hydrochloric acid was purchased from PIOCHEM, Giza, Egypt. Recombinant human PD-1/FC chimera (PD-L1) and horseradish peroxidase-conjugated mouse anti-human IgG4 were kindly supplied by Bristol Myers Squibb, Devens, MA, USA. Supplies of 50% casein blocker, TMP substrate, and stop solution were purchased from Thermo Fisher Scientific, Waltham, MA, USA. The A549cell line was purchased from Vacsera, Cairo, Egypt. Jurkat T cells (leukemia cells) were obtained from the American Type Culture Collection (ATCC), Manassas, VA, USA. Penicillin-streptomycin Ab (10,000 U/mL), glutaMAX, and ready-made Roswell Park Memorial Institute (RPMI) 1640 medium were purchased from Gibco, New York, NY, USA. The Cell Titer 96^®^ Aqueous One Solution Cell Proliferation Assay (MTS) was purchased from Promega, Madison, WI, USA. Nivolumab was kindly supplied by Bristol-Myers Squibb, Princeton Pike, NJ, USA. Tetramethylbenzidine (TMP) substrate solution was obtained from MaxiSorp^TM^, Thermo Fisher Scientific, Waltham, MA, USA.

### 4.2. Methods

#### 4.2.1. Preparation of Plain GNPs

The procedure used to prepare GNPs involved minor changes to the double desolvation method described by Coester and colleagues [77], with optimization of independent factors such as the amount of cross-linker, cross-linking times, and stirring speeds. To prepare the nanoparticles, an initial desolvation step was performed. This step involved precipitating the high-molecular-weight gelatin chains, where gelatin was dissolved in 25 mL of water, followed by the addition of 25 mL of acetone. The purpose of this precipitation process was to isolate and separate these specific gelatin chains for further use in nanoparticle formation. The low-molecular-weight gelatin chains present in the dispersion supernatant were discarded [78]. In the second desolvation step, which plays a crucial role in the formation of GNPs, the gelatin dispersion was titrated with acetone dropwise at 40–45 °C to form the nanoparticles. To stiffen the GNPs, different concentrations of cross-linker glutaraldehyde (GA) (12.5, 25, and 50% *v*/*v*) were added to the nanoparticle dispersion to initiate intra-particular cross-linking for different time intervals (8, 16, and 24 h). At the end of a given time interval, the GA cross-linking effect was stopped by adding glycine (751 mg/100 mL) to block its carboxylic groups. The mixture was stirred at 600 rpm or 1200 rpm. After three centrifugation/redispersion cycles in purified deionized water, the GNPs were purified. The composition of different formulations is revealed in Table 1.

#### 4.2.2. Nivolumab Loading into GNPs

The process of incorporating nivolumab into GNPs involved the solubilization of a measured amount of the drug (10 mg) into deionized water (25 mL), forming an aqueous solution. The formed solution was employed to redisperse the gel-like precipitate formed as part of the initial desolvation phase to guarantee complete dispersion and binding between nivolumab and GNPs.

### 4.3. Characterization of the Prepared GNPs

#### 4.3.1. Determination of Particle Size, Particle Size Distribution, and Zeta Potential of the Prepared Nanoparticles

Photon correlation spectroscopy was used to measure the particle size (PS) and size distribution by measuring average volume diameters and polydispersity indexes (PDIs) using a dynamic light scattering (DLS) particle size analyzer (Zetasizer Nano ZN, Malvern Panalytical Ltd. Worcestershire, UK) at 173° at 25 °C. Samples were diluted with deionized water before measurements.

The zeta potential was measured using the same equipment with folded capillary zeta cells. All data are described as the mean of triplicates ± standard deviation.

#### 4.3.2. Determination of Nivolumab Entrapment Efficiency (% EE) in GNPs

The drug entrapment efficiency was determined by an indirect method [79], where the free (unloaded) drug was measured in the clear supernatant after the separation of nanoparticles using a combined ultracentrifugation at 10,000 rpm for 20 min using a freeze centrifuge (Sigma 2-16 KL, Osterode, Germany). Then, 500 µL of supernatant was analyzed using a UV spectrophotometer (Shimadzu, Kyoto, Japan) at λmax equal to 280 nm. The results obtained were then compared against pre-constructed calibration curve measurements to ensure consistency, using solutions obtained from corresponding plain formulations as blanks. Finally, entrapment efficiency was calculated as the percentage of drugs entrapped by utilizing the following equation [9,37,80]:

(1) $$\%EE=\frac{Total\;amount\;of\;protein-Total\;protein\;amount\;in\;supernatent}{Total\;amount\;of\;protein}*100$$

#### 4.3.3. Transmission Electron Microscopy

The advanced TEM (JEM-1400 device, Tokyo, Japan) was utilized to conduct a thorough investigation into the intricate morphology and utmost homogeneity of the prepared nivolumab-loaded GNPs. To enhance the conductivity of the GNP suspension, the sample was carefully mounted onto a carbon-coated copper grid, and then allowed to dry at ambient room temperature before precise examination at a high voltage of 200 kV to keep the surface conductive; consequently, the electrons could be captured more readily, leading to improved image resolution [9,37].

#### 4.3.4. In Vitro Release Study of the Nivolumab-Loaded GNPs

An in vitro release study was conducted to determine the kinetics of nivolumab release. GNPs were incubated in 20 mL of PBS media at two different pH levels: pH 7.4 (physiological pH) and pH 6.8 (cancer cells). The temperature was maintained at 37 °C with continuous stirring at a speed of 550 rpm [81]. This experimental setup ensured the effective dispersion of pellets and allowed for accurate measurements throughout the duration of the study. To monitor the rate of nivolumab release over time, a sample volume of 500 microliters was withdrawn at each interval period (0.5, 1, 2, 4, 6, 8, 12, 24, 48, and 72 h), which would be instantly replaced with fresh buffer solution. After each withdrawal period, samples underwent centrifugation at high speeds, reaching approximately 120,000 rpm for ten minutes before measuring the concentration of released nivolumab present in the supernatant using spectrophotometric methods specifically designed by Shimadz, Kyoto, Japan, at λ_max_ of 280 nm.

#### 4.3.5. Determination of the Binding Activity and Relative Potency of Nivolumab-Loaded GNPs

**Figure 11:**
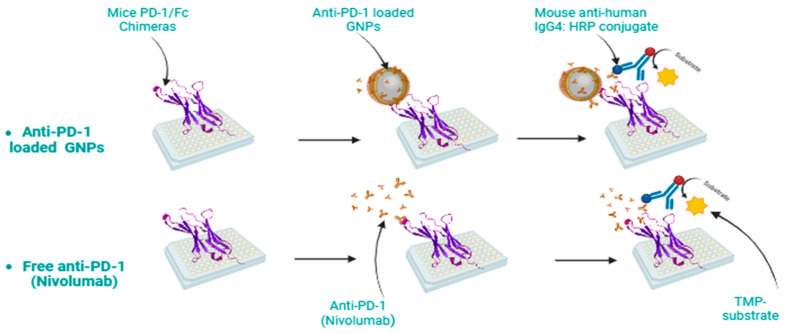
Schematic illustration of ELISA analysis to determine the binding of anti-PD-1 to its receptor.

Relative potency and binding activity rely on the assumption that both the reference and test material are biologically comparable, with the additional expectation that the behavior of the test material aligns closely with a concentration or dilution of the standard [82]. To comprehensively analyze the binding affinities between nivolumab and the PD-1 receptor, a rigorous investigation was carried out using three distinct ELISA assays (Figure 11). These assays were performed to compare the reference values of nivolumab solution with those obtained from nivolumab-loaded GNPs. This approach was employed to gain deeper insights into the interaction dynamics between nivolumab and PD-1, as illustrated in Figure 1.

First, a 96-well flat-bottom immuno-plate was coated overnight with a concentration of Mice PD-1/Fc Chimeras (0.5 µg/mL). After three washes using PBS and 0.05% Tween20, the plate was blocked with 50% casein blocker for two hours at 37 °C. Next, serial dilutions were prepared for nivolumab-loaded GNPs samples and free nivolumab, starting from a concentration of 1 µg/mL. At least eight dilutions were made for each sample to induce improvements, including the introduction of parallelism. These diluted samples were then added to the wells on the same immuno-plate mentioned earlier, followed by another round of washing with PBS and Tween20 solution. The plates were incubated with mouse anti-human IgG4:HRP conjugate at a ratio of 1:5000 for one hour. Then, a tetramethylbenzidine (TMP) substrate solution was added to start the reaction. The reaction was stopped after ten minutes using TMP stop solution, and the reading of the plate in dual wavelength mode was recorded. The color was quantified using a microplate spectrophotometer at 450 nm with a 650 nm blank subtraction [83,84].

Data analysis was conducted using a 5-parameter logistic curve fit using GraphPad Prism version 8, specifically, an asymmetrical curve. The triplicate absorbance values for both reference and test samples were plotted (Y) with respect to the log scale concentration (X) of nivolumab. This resulted in generating an 8-point dose-response curve, which was then fitted to a 5-parameter logistic equation. Due to the complexity of the biological system, a non-linear dose–response curve relationship was derived, requiring consideration of multiple parameters during analysis.

Asymmetrical dose–response curves can be described by various equations, one of them being the Richards version [72], which is integrated into Prism’s ‘Dose-response—Special’ feature. The characteristic sigmoidal shape of the curve is generated by fitting the data across a range of concentrations of the tested material. At low concentrations, there is not enough drug to stimulate a measurable biological response, while at high concentrations, the receptor becomes saturated and there is no additional response. In the middle of the curve, the relationship becomes linear, and then the EC_50_ and goodness of fit can be calculated. Each response variable comprised a separate analysis, and a separate dose–response curve was fitted for each population. Using the five-parameter log-logistic equation, this equation is also referred to as the generalized Hill equation:

(2) LogXb=LogEC50+(1/Hillslope)∗Log((2^(1/S))−1)

Numerator = (Top − Bottom), Denominator = (1 + 10^(LogXb − X) ∗ Hillslope))^S, Y = Bottom + (Numerator/Denominator). This equation assumes that X has been entered as (or transformed into) the logarithm of concentration, Y is the response, (S and Hillslope) is the asymmetry parameter, and LogXb is the inflection point, which is distinct from the EC_50_. The relative potency can be calculated by dividing the EC_50_ of the sample over the EC_50_ of the reference [85]. The relative potency estimate is valid only when the dose–response curves for the reference and test substances are statistically parallel [85]. There are two main approaches to parallelism testing in research. One assumes that the datasets are parallel (null hypothesis, denoted H0), while the other examines evidence against this assumption and considers non-parallel data (alternative hypothesis, denoted H1) [86,87]. To determine statistical significance, a model was fitted separately to our test and reference datasets. The comparison is based on a *p*-value, which measures the likelihood of seemingly non-parallel datasets occurring due to random variability in the data. The *p*-value is derived from statistical analysis and involves three common methods for assessing parallelism.

Different approaches are used, like the F-test, the X2-test, and an equivalence test [87], The F-test, recommended by the European Pharmacopoeia guideline [88], is a classical statistical test used to compare two models. It assumes that the variance remains constant across all groups in the assay. In this approach, the null hypothesis posits that both standard and test products are similar [86]. A significance level of 0.05 is typically chosen [89]. If the calculated *p*-value falls below this threshold value, it indicates a failure to meet parallelism requirements in the assay result. Conversely, if there is no significant evidence pointing towards non-parallel datasets above this set boundary value, then a parallel assumption is proposed [90].

### 4.4. In Vitro Cytotoxicity of Different Cell Groups

To assess the in vitro cytotoxicity of the formulation compared to the free drug, MTS [3-(4,5-dimethylthiazol-2-yl)-5-(3-carboxymethoxyphenyl)-2-(4-sulfophenyl)-2H-tetrazolium] was utilized. This salt can be transformed into formazan products by viable cells, which are soluble in cell culture medium. Two different cell line models were employed for this study; A549 cells, known for their expression of the PDL-1 receptor and commonly used as a lung cancer model [64,75,91], and a co-culture system consisting of A549 cells combined with Jurkat cells. The Jurkat cell line is an immortalized T lymphocyte cell line that expresses the PD-1 receptor [64]. Initially, it was important to determine the viability of cells over various time intervals (24, 48, 72 h) using non-loaded GNPs to assess their ability to display any significant cytotoxicity in tumoral cells by measuring cell viability using the following equation [51]:

(3) $$cell\;viability\;rate=\left(\frac{sample\;absorbance}{control\;absorbance}\right)*100$$

Subsequently, the IC_50_ values for both the free drug and drug-loaded GNPs were determined using non-linear regression analysis employing four parameters, including a variable slope.

#### 4.4.1. Determination of IC_50_ of Free Nivolumab and Nivolumab-Loaded GNPs on the A549 Cell Line

The A549 cells were cultured in RPMI-1640 medium supplemented with 10% fetal bovine serum and 1% penicillin–streptomycin, ensuring optimal conditions for growth. Careful monitoring every two days was conducted on the flasks containing these cells prior to subculturing using trypsin-Versene, therefore guaranteeing continuous cellular proliferation [75]. To evaluate the IC_50_ of nivolumab, a serial dilution starting from 3.482 µM down to 0.109 µM was prepared by adding free nivolumab and nivolumab-loaded GNPs into A549 cell cultures over time intervals of 24 h, 48 h, and 72 h within a CO_2_ incubator (Heraeus HERA cell CO_2_ incubator-RS232). Post-treatment analysis involved counting viable cells at each time point relative to negative control cultures that did not receive any intervention with nivolumab. This approach allowed us to determine cytotoxicity expressed as a percentage relative to negative control cultures grown without nivolumab.

#### 4.4.2. Determination of IC_50_ of Free Nivolumab and Nivolumab-Loaded GNPs on the Co-Culture of A549 Cells with Jurkat Cells

The effect of Jurkat cells in combination with free nivolumab and nivolumab-loaded GNPs on A549 cells was investigated, where 50 μL A549 cells was seeded at 1 × 10^5^ cells/well in a flat-bottomed 96-well plate. Subsequently, 50 μL of effector Jurkat cells at a density of 1 × 10^4^ cells/well was co-cultured at the selected effector-to-target ratio (E:T), which was found to be 1:10 [59] for the incubation period (24, 48, and 72 h), and treated with nivolumab solution or drug-loaded GNPs. At the end of the incubation period, effector cells were discarded, and the plates were washed with PBS several times; then, the target cells were stained with MTS [75]. Absorbance was measured at 490 nm using a Tri- star LB942 multimode plate reader. Percent cytotoxicity was determined and IC_50_ was calculated using the following equation [52,92,93]:

(4) $$\%\;\mathrm{Cytotoxicity}=100-\frac{absorbance\;of\;treated\;target\;cells\;average}{absorbance\;of\;untreated\;target\;cells}*100$$

### 4.5. Stability Testing

To ensure the stability of nivolumab-loaded GNPs during storage, a series of tests were conducted at specific intervals (1, 3, and 6 months). The objective was to determine whether the drug’s biological activity remained intact over time. The binding rate of the selected formula was assessed using ELISA. A 96-well immuno-plate was coated with Mice PD-1/Fc Chimeras and blocked with casein blocker. The drug solution, a selected formula of drug-loaded GNPs, and plain GNPs were added to separate wells. The plates were then incubated with mouse anti-human IgG4:HRP conjugate, and the reaction was started by adding TMP substrate solution. After stopping the reaction with TMP stop solution, the plate reading at 450 nm (with blank subtraction) was recorded using a microplate spectrophotometer in dual wavelength mode [46]. The binding rate as a percentage of the absorbance of the tested group over the reference group can be calculated by the following equation [53]:

(5) $$Binding\;rate\;\%=\frac{Abs\left(test\right)-Abs(background)}{Abs\left(control\right)-Abs(background)}\times 100$$

These investigations allowed us to monitor any changes or degradation that may occur during storage, thus ensuring that the drug maintains its effectiveness throughout the storage. By carefully assessing these parameters at regular intervals, we can confidently demonstrate that our formulation can maintain the integrity and therapeutic potential of nivolumab [65].

### 4.6. Statistical Analysis

All data are represented as the means of three replicates ± standard deviation. Statistical analysis was performed using GraphPad Prism 8 (GraphPad Software, Inc., San Diego, CA, USA). The binding activity was determined using a 5-parameter logistic curve fit, and the parallelism was determined using the F-test to compare fits with alpha equal to 0.05. The normality distribution of the data was tested by a non-linear regression normalized dose–inhibitory response curve. The cytotoxicity comparisons were performed by a 2-way ANOVA, with Tukey’s test used for multiple comparisons between groups depending on the assay; *p*-values are indicated, where an appropriate *p*-value of *p* < 0.05 was considered statistically significant, also using a one-way ANOVA [62].

## Figures and Tables

**Table 1:** Effect of different formulation variables on the particle size, zeta potential, and polydispersity index of the prepared plain gelatin nanoparticles.

Formula Code	GA%	CLT(h)	Stirring Speed (rpm)	PS (nm)	PDI	ZP (mV)
GNP1	25	8	600	257.5 ± 11.47	0.034 ± 0.032	20.9 ± 0.20
GNP2	50	8	600	158.6 ± 1.08	0.041 ± 0.01	21.7 ± 0.15
GNP3	25	16	600	293.5 ± 4.80	0.031 ± 0.02	22.2 ± 0.41
GNP4	50	16	600	285.5 ± 2.38	0.064 ± 0.02	21.1 ± 0.15
GNP5	25	16	1200	360.6 ± 10.92	0.302 ± 0.03	−6.8 ± 0.38
GNP6	12.5	16	600	Aggregate formation		
GNP7	25	>24	600	Aggregate formation		

GA: glutaraldehyde percentage, CLT: cross-linking time.

**Table 2:** Results of the three independent ELISA studies of the binding activity of the optimum nivolumab-loaded GNPs when compared to free nivolumab as a reference using non-linear regression with five parameters.

Best-Fit Value	Run 1	Run 2	Run 3
EC50	0.039 ± 0.592	0.057 ± 0.596	0.057 ± 0.594
Relative potency (binding activity)	99.42%	95.42%	92.14%
Goodness of Fit (R^2^)	0.997	0.995	0.997
Hillslope	2.698	2.558	2.061
S	0.371	0.477	0.656
Top	3.169	2.752	2.823
Bottom	0.085	0.149	0.143
Log EC_50_	−1.406	−1.245	−1.247

## Data Availability

Data would be available upon request.
